# Experiences of peer counsellors in supporting exclusive breastfeeding in southern India: An exploratory qualitative study

**DOI:** 10.1111/1471-0528.17615

**Published:** 2023-08-02

**Authors:** Umesh S. Charantimath, Roopa M. Bellad, Niranjana Mahantashetti, Chandrashekar C. Karadiguddi, Geetanjali M. Mungarwadi, Patricia J. Kelly, Tony Ma, Shivaprasad S. Goudar, Richard J. Derman

**Affiliations:** 1 KLE Academy of Higher Education and Research, Jawaharlal Nehru Medical College, Belagavi, Karnataka, India; 2 Thomas Jefferson University, Philadelphia, Pennsylvania, USA; 3 Benten Technologies, Inc., Manassas, Virginia, USA

**Keywords:** exclusive breastfeeding, experiences, peer counsellors, qualitative research

## Abstract

**Objective:**

Peer counsellors are effective in addressing a variety of health challenges, including exclusive breastfeeding (EBF). Providing education and support from a person of similar background and experience has been an important adjunct to the practice of health workers for the past 50 years.

**Design:**

It is an exploratory qualitative study.

**Population or Sample:**

Twenty-two peer counsellors.

**Setting:**

In-depth Interview in the community.

**Methods:**

To better understand the experiences of these important health workers, we conducted qualitative interviews with 22 peer counsellors who participated in a research study in Belagavi District, Karnataka, India. Transcripts of the interviews were organised and assigned codes by the research team.

**Main outcome measures:**

Experience of Peer counsellor’s role in the community to improve breastfeeding practices.

**Results:**

Peer counsellors had a good understanding of the larger study and of their role. Analysis of the transcripts identified three themes: personal satisfaction; the effect on the larger circle of family and community; and ideas for future programming. The positive experiences and the ability of peers to be trained in counselling women around EBF support their use in breastfeeding support and other areas of health education.

**Conclusions:**

The women from the community who served as peer counsellors were enthusiastic and satisfied about their work, which provided them with opportunities to do meaningful community work outside of their household routines. Use of the peer counsellor model to deliver a specific objective like improvement in EBF rates, immunisation or mental health in integration with healthcare providers can help in achieving desired goals.

## 1 ∣ BACKGROUND

Peer counsellors (PCs) work successfully in community settings to address problems as diverse as suicide prevention, HIV disease, postpartum depression, substance use disorders and chronic diseases such as diabetes and asthma.^1^ Providing education and support from a person of similar background and experience has been an important adjunct to the practice of health workers for the past 50 years.^2^ Begun in the 1970s as an adjunct to the mental health consumer movement, PCs are now a recognised component of healthcare systems. Their familiarity with the health issue they are addressing allows them to provide services that are beyond the reach of many trained and educated health workers who have not experienced the same day-to-day issues.

Programmes supporting exclusive breastfeeding (EBF) have recognised the value of PCs. One older review identified 11 randomised controlled trials and concluded that peer support increased the duration of exclusive breastfeeding in low- and middle-income countries.^3^ PCs have been able to provide antenatal education about the importance of EBF and to make support available for breastfeeding challenges outside of that provided by midwives, nurses and physicians during the labour and delivery process. Such education and support has proved to be critical in helping individual women to practise EBF and to increase country-wide rates of this critical health practice.^4^

However, the actual experiences of PCs have not been explored. How did the training and work with breastfeeding mothers impact them? What challenges did they experience? What can EBF programmes using PCs learn from their experiences? The goal of the current exploratory work was to begin to address these questions.

### 1.1 ∣ Description of the peer counsellor breastfeeding intervention

With the overall aim of assessing a community-based intervention using PCs to improve rates of EBF, 25 women with personal breastfeeding experience were recruited to serve as PCs in the BEST4Baby intervention. Women participated in a 3-day training that consisted of interactive lectures, demonstrations and role-playing to provide both knowledge and self-efficacy around supporting EBF.^5^ A novel component of the intervention was the incluclusion of a mobile health (m-Health) application, based on a World Health Organization curriculum adapted to the local culture.^6^ All 25 women successfully met knowledge and counselling competencies and were each assigned five to seven pregnant women who had consented to receiving breastfeeding counselling. Two counselling sessions were scheduled prior to delivery and seven occurred during post-delivery home visits (within 3 days of delivery, on days 7 and 14, and at 1, 2, 4 and 6 months after delivery). The PCs were paid a small stipend for each visit and had their transportation costs reimbursed. Educational content for each visit and for the m-Health app were developed from extensive community work assessing barriers and facilitators to EBF.^7^ The final intervention content, as well as all traning materials, were reviewed by the study's advisory board of EBF experts.

## 2 ∣ METHODS

This exploratory qualitative study used individual, semistructured interviews to understand the experiences of PCs who participated in a community-based research study. The goal of the study was to assess the impact of the PC intervention on EBF. Details about the larger study are provided elsewhere.^6^ Approval to conduct the study was obtained from the Institutional Ethical Committee approval at the KLE Academy of Higher Education and Research (formerly known as KLE University) in India and the Thomas Jefferson University in the USA. All participants provided signed informed consent.

### 2.1 ∣ Setting/sample

The study took place in the Belagavi District, Karnataka, India. PCs were selected through written notices posted at five primary health centres. Criteria for selection included breastfeeding experience, a minimum of 10 years of education, and familiarity with the use of a smart phone and apps. Of the 25 PCs who began work with the research project, 22 completed the study and participated in the interviews reported here.

### 2.2 ∣ Data collection

Faculty members trained in qualitative methods who also had familiarity with PCs and knowledge of the larger study conducted the interviews. PCs were contacted in February 2020 immediately after the data collection portion of the study ended. Interviews which ranged from 30 to 45 minutes in length were conducted by telephone due to COVID restrictions. The interviews were conducted in Kannada, the local language with which the counsellors were familiar. All interviews were audio-recorded. The 21-item interview guide, which assessed the experiences of PCs, was based on the work of Mihrshahi et al.^8^ Two interview questions were added to assess understanding ofthe larger study and understanding of the role of the PC. To assess *understanding of the larger study*, PCs were given one point for mentioning any of the following seven components of the overall study:

1. Training and capacity building of PC in the community;
2. Improving the rate of EBF;
3. Increasing the knowledge and importance of breastfeeding;
4. Avoid giving any fluids or liquids other than breast milk and avoiding formula feeds until 6 months;
5. Emphasising appropriate breast care and nutrition to mothers;
6. Use of m-Health in counselling;
7. Assessing the development of the baby by regular weight monitoring in seven postnatal visits;
8. Problem finding and resolving capacity of PC in breastfeeding.

PCs who mentioned in their interviews five or more components were considered to have high understanding, three–four components as having moderate understanding and one-two as having poor understanding.

To assess *understanding of the PC role* in the study, PCs were given one point for mentioning any of the following eight components of the overall study:

1. Counselling about breastfeeding;
2. Identify number of scheduled visits;
3. Identify and resolve problems of breastfeeding;
4. Demonstration of breastfeeding using the breast model and baby doll;
5. Demonstration of m-Health components;
6. Entry of data in m-Health application;
7. Confidence in counselling;
8. Building good relation with family members, healthcare provider and community members.

PCs who mentioned more than six responses for the abovementioned components were considered to have high understanding, four–six as having moderate understanding and fewer than four as having poor understanding of the role.

### 2.3 ∣ Data analysis

Audio-recordings of the interviews were transcribed verbatim and translated from Kannada to English. Transcripts were checked against the original recordings for accuracy and organised using NVIVO 12 software. Two independent investigators experienced in qualitative research open-coded the transcripts and developed an initial list of codes based on the topic areas of the interviews. Common themes were independently identified by one author and confirmed by a second author. Codes were reviewed with the research team. Final codes were chosen and discrepancies were resolved. The principal and second authors used the codes to identify relevant quotes. Themes and quotes were reviewed with all authors to arrive at consensus.^9^

## 3 ∣ RESULTS

The 22 PCs interviewed for this study had an average age of 30.1 years (range 20–43); 19 (86.4%) were currently married (mean 11.9 years) and had an average of 2.1 children; 41% had a 10th grade eduction and 17 (77%) had some previous experience working in social services. These and other demographics are described in Table 1.

Almost all of the PCs (*n* = 20, 91%) had a moderate to high understanding of the larger study; an equal number had a moderate to high understanding of the PC role in the study (Table 2).

Three themes were evident from the interviews: personal satisfaction; the effect on the larger circle of family and community; and ideas for future programming.

### 3.1 ∣ Personal satisfaction

PCs expressed their satisfaction with their role in a variety of ways: pride in their knowledge and skills; being able to resolve breastfeeding problems; the ability to resolve challenges to their role; and time management.

Almost every PC interviewed, expressed pride in their knowledge and skills in helping breastfeeding mothers:

I am very much happy and satisfied with the job as a peer counsellor in the study. We were not having much knowledge about breastfeeding before we entered into this study. Even when I delivered my baby I had not much knowledge about breastfeeding … Now I got an opportunity to counsel 4–5 mothers about breastfeeding I am happy that I gave the knowledge about breastfeeding to 5–6 mother which I had not got when I was pregnant and delivered my baby. The mothers whom I counselled are also happy and they told me that ‘My baby is healthy and fine because you counselled me about breastfeeding. We had the wrong belief that by breastfeeding the baby I would lose my beauty. But breastfeeding is very useful and helpful for the growth of the baby’, that also made me very happy. It was very useful for the mothers also.

Personally, I felt that it is a sort of social service work; and helping a baby to grow healthily and without any diseases. Many mothers were not aware of the benefits of breast milk. So, this study helped many mothers in the community.

Satisfaction was evident in the counsellors’ confidence in being able to resolve breastfeeding problems:

They told me that the breasts have become tight and milk is not coming. I had been trained to remove the milk during the training. I removed the milk and showed them … The training helped me.

I can deal with problems. We also have a book with information. In case I forget anything, I look into the book and give them information.

The problems that mothers face are, they don't know how to breastfeed the baby at the outset, especially those with first delivery. They can't sit properly, can't feed the baby. They say the nose may become blunt and hence do not feed the baby properly… I further explained and make her feed the baby.

Because I have undergone the training, I have the courage to give information. I will clarify any doubt.

Satisfaction was also seen in their ability to resolve challenges in their roles as peer counsellors.

Initially I had a problem in one mother's house. They were not listening to me with attention.

They were not bringing the baby to take its weight. But after two visits they started cooperating with me and they started themselves calling me for visits.

One member of a mother's family objected us. But when we explained to them about the study and our job they got convinced and they understood that this study is for their benefit and after that they did not object.

I did not go into counselling immediately after going into their house. I was spending some time talking with them casually for some time to take them into my confidence. I used to start my actual counselling session and there used to be no disturbance into the counselling session.

A final area of satisfaction expressed by the PCs was in time management:

I used to note the dates, check the tablet and I kept the visit related books and checked information about the woman whom I had to visit. I used to prepare myself by going through the content in the tab. For this I have never faced any problem even from my family since I am residing at my mother's place. Even though I have two children, one studying in fifth grade, the other is a 4-year-old girl, I used to complete my personal work well in advance. So, I had no problem.

I used to go for visit when I was free from my housework and also when the mothers were free from their work. I used to plan the visit by discussing with them on phone.

I did not feel my job as workload. I used to go for visit in the morning after my children had been to the school and I used to come back home before the children used to come back from the school.

### 3.2 ∣ Effect on larger circle of family and community

The work of the PCs had an effect on both their families and their communities. Almost every woman mentioned the support provided by their families:

My husband gave me support especially in going to the visit to the mothers. He used to take me on his two-wheeler for the visits.

My husband supported my work because it is a social work. He himself used to take me on his vehicle to complete some visits when it was raining on that day.

My brother used to accompany me.

My family members gave all the support to me to do my work as peer counsellor.

Whenever I was out for a visit my mother-in-law used to take the care household work.

Community members knew about the PC's role and made use of her skills.

Even now also after the study is over the mothers take my advice in breast feeding.

Other mothers also asked me to counsel them.

The people in the community also liked my job and appreciated it. Other mothers also were requesting me to counsel them.

The counselling we gave to the mothers was understood by their relatives, neighbours and family members also who were present during our counselling.

The family members of the enrolled mother were also interested to learn from me and were sitting attentively while I was counselling the mother.

My family members gave me support to do my work as peer counsellor. My husband and my mother-in-law used to remind me about the days of visits sometimes, so that I should not forget any visits being done.

### 3.3 ∣ Ideas for future programming

Several PCs made concrete suggestions about what researchers might do in future work. While they agreed that the app was especially useful for mothers who were illiterate, they wanted more tools. Specifically, they requested a greater variety of videos, and ones that were in all of the local languages, so they did not have to translate the information for the few mothers who did not speak Kannada. Finally, they requested specific videos that, in addition to telling mothers not to give honey or gutti to babies, provided information about the negative effects of these products.

PCs made concrete suggestions about the need for additional visits at around 3–6 months postpartum, a time when women might be considering giving water to their babies. Finally, they wanted to do more visits. Several mentioned that they could handle a larger case load:

The visits are very few in a week or in a month … So we feel bored. We will feel nice if we have visits every day.

## 4 ∣ DISCUSSION

In this study, PCs were satisfied with the knowledge they had obtained from training and with their ability to apply this knowledge in support of a social good. These findings are similar to those found in a study conducted in Dhaka by Mihrshani and colleagues.^8^ Witnessing the improvement in breastfeeding practices and social support in the society, the PCs felt a sense of emotional satisfaction and were confident about their work, similar to findings reported by PCs in other fields.^10^ The counselling resulted in a two-way flow of support: the mothers benefitted in their ability to care for their babies and the PCs in their satisfaction in observing the mothers providing the skilled care.^11^ The PCs’ sense of contribution and skills is important because many of them had minimal education and yet were effective in providing the knowledge necessary for successful breasteeding.^12^

The effect of education in community settings resulted in an impact beyond the immediate trainees. The support provided by family members and the recognition of the role of the PCs in the community was also present in the interviews with PCs in Dhaka.^8^ While it may not be possible for researchers to capture the full extent of the training impact, it is clear that their knowledge and skills continue to be shared.

## 5 ∣ LIMITATIONS

Limitations of the study include that the interviews were conducted by telephone due to COVID-19 pandemic restrictions, so non-verbal expressions could not be noticed. Also, the interviews were conducted by the research team which provided the training and supervision of the study, so negative responses might have been minimised.

## 6 ∣ CONCLUSION

Women from the community who served as PCs were enthusiastic about their work, which provided them with opportunities to do meaningful community work outside of their household routines. The training increased their knowledge about breastfeeding, and improved communication skills; the work provided them with new friends and with recognition of an important role in their community. Physicians and nurses are often overburdened with healthcare activities as a result of limited staff and the many health needs of the community. Use of the PC model to deliver a specific objective such as improvement in EBF rates, immunisation or mental health in integration with healthcare providers can help in achieving desired goals. Use of technology such as m-Health applications has the potential to improve health-seeking behaviour and knowledge about various health issues, and enhance the living standards of the community.

## Figures and Tables

**TABLE 1:** Socio-demographic details of peer counsellors (*n* = 22).

Demographic	*n*/%
Age	
20–25 years	2/10%
26–30 years	11/50%
31–35 years	7/31.8%
>35	2/9%
Marital status	
Married	19/86.4%
Widow	2/10%
Divorced	1/4.5%
Years married (mean 11.9)	
<5 years	1/4.5%
6–10 years	8/36%
11–15 years	8/36%
16–20	5/22.7%
Number of children	
1	3/13.6%
2	13/59.1%
3	5/22.7%
4	1/4.5%
Age of youngest child	
< 5 years	6/27%
6–10 years	10/45%
11–15 years	6/27%
Education level	
8-10th grade	3/13.5%
10th grade completed	9/41%
Pre-university (11 and 12 grade)	7/32%
Bachelor's degree	3/13.5%
Experience using smart phone (mean 20.4)	
< 12 months	6/27%
13–24 months	13/69%
>24 months 25–48 months	3/13.5%
Previous social service experience	17/77%

**TABLE 2:** Understanding of study and role of PC.

Understanding of overall study	*N*/%
High	6/27%
Moderate	14/64%
Poor	2/9%
Understanding of peer counsellor's role in study	
High	4/18%
Moderate	16/73%
Poor	2/9%

## Data Availability

The data is available for sharing on request.
